# Exploring the Effectiveness of “Four‐Point” Configuration Cannulated Compression Screw Implantation Protocol for the Fixation of Pauwels Type III Femoral Neck Fractures in Adults

**DOI:** 10.1111/os.70377

**Published:** 2026-07-17

**Authors:** Yujie Li, Zhenyu Wen, Zhang Jian, Naiqiang Zhuo, Shi Shen

**Affiliations:** ^1^ Department of Joint Surgery People's Hospital of Leshan Leshan China; ^2^ Department of Orthopedics Affiliated Hospital, Southwest Medical University Luzhou China

**Keywords:** biomechanical analysis, femoral neck fracture, femoral neck system, finite element analysis, inverted cannulated cancellous screw, Pauwels III

## Abstract

**Background:**

Pauwels type III femoral neck fractures (P3FNFs) are one of the common types of femoral neck fractures (FNF) caused by high‐energy violent factors. This study aims to evaluate the efficacy of inverted cannulated cancellous screw (ICCS) assisted transversal cannulated screw (TCS) perpendicular to the fracture line in young adults with Pauwels type III femoral neck fractures.

**Methods:**

Construct an internal fixation‐fracture model through finite element analysis (FEA) and biomechanical test (BT) to simulate the stress environment of the human hip joint, and utilize the femoral neck system (FNS) model as a control to predict the biomechanical properties of ICCS + TCS. From January 2023 to December 2024, 21 patients with P3FNFs treated with ICCS + TCS were collected, and their recent radiographic and clinical outcomes were evaluated. The experimental data underwent statistical analysis using one‐way ANOVA and independent samples *t*‐test.

**Result:**

ICCS + TCS exhibits biomechanical trends in FEA and BT that are essentially consistent with those of FNS. ICCS + TCS has stronger torsional stiffness and lower overall deformation, But the resistance to vertical shear force is weaker than FNS. All patients in this study successfully completed the surgery without complications such as vascular or nerve injuries. The follow‐up period ranged from 8 to 25 months. The hip joint flexion‐extension ROM, internal–external rotation ROM, and Harris score all significantly increased (*p* < 0.05). The shortening length of the femoral neck was 3.87 ± 0.44 mm.

**Conclusion:**

ICCS + TCS exhibits biomechanical trends compared to FNS; meanwhile, it has stronger torsional resistance and load‐bearing deformations. Simultaneously, ICCS + TCS has demonstrated favorable outcomes in clinical treatments. This study offers a novel approach for the treatment of P3FNFs.

## Graphical abstract

This experiment explored the biomechanical performance of ICCS + TCS in the fixation of Pauwels type III femoral neck fractures in young adults and results indicate that ICCS + TCS exhibit biomechanical properties similar to FNS in the treatment of P3FNFs while possessing stronger torsional stiffness and lower overall deformation.

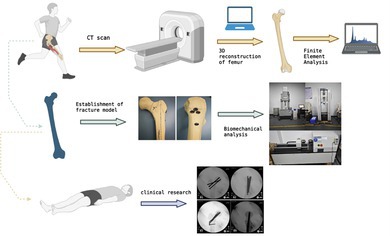

## 1 Introduction

Femoral neck fracture is a relatively common disease among traumatic injuries of the limbs in orthopedics. Currently, FNF accounts for 3.58% of all fractures in the body and 50% of all hip fractures [1, 2, 3]. Pauwels type III is one of the common types of femoral neck fractures caused by high‐energy violence. This type of fracture is a special type of unstable fracture, characterized by its unique anatomical location, surrounding blood supply, and high shear and rotational forces at the fracture site, leading to a high incidence of postoperative complications [4, 5]. Therefore, the key conditions for treating Pauwels type III femoral neck fractures include anatomical reduction and rigid fixation of the fracture ends.

For the clinical treatment of Pauwels type III femoral neck fractures, the selection of implant devices includes hollow screws, dynamic hip screws, medial support plates, proximal femoral nail systems, etc. However, the optimal internal fixation method for P3FNFs has not yet been fully determined [6]. The most widely used treatment for femoral neck fracture in China is inverted cannulated cancellous screw [7]. However, existing studies [8, 9, 10] have reported that the use of ICCS for P3FNFs yields poor results, with a postoperative complication rate of 46.7% and a postoperative femoral head necrosis rate that can reach 52.9%.

The femoral neck system (FNS) developed by Stoffel is a novel internal fixation device that began to be applied in clinical treatment around 2017 [11]. It possesses excellent capabilities in resisting shear forces, resisting torsion, preventing screw backout, and dynamically compressing fracture ends. Numerous studies [12, 13, 14] indicate that compared to ICCS, FNS demonstrates superior biomechanical performance and applicability in the treatment of P3FNFs.

Researches [15, 16] have shown that inserting a cannulated compression screw (CCS) parallel to the long axis of the femoral neck, based on ICCS, and forming a rhombic arrangement can effectively enhance the resistance to vertical shear forces and improve the mechanical properties of the femoral neck. Based on the rhombic configuration of hollow screws, this article modifies the placement of the fourth hollow screw by inserting it into the lateral wall of the femur in a direction perpendicular to the fracture line.

Therefore, the purpose of this study is to (i) propose a “Four‐point” configuration cannulated compression screw implantation protocol for the femoral neck of Pauwels type III in young adults, (ii) verify the biomechanical performance of the surgical protocol using finite element analysis and in vitro biomechanical testing, and compare it with FNS, (iii) evaluate the clinical effectiveness of the four‐point fixation protocol through clinical trials, and (iv) explore whether it can become an alternative clinical treatment for the femoral neck of Pauwels type III in young adults, laying a theoretical foundation for future clinical promotion.

## 2 Materials and Methods

### 2.1 Finite Element Analysis

#### 2.1.1 Models Establishment

Femur computed tomography (CT) data was obtained by scanning the femur of a healthy, 20‐year‐old male with no specific diseases or past medical history (weight: 70 kg, height: 178 cm, BMI 22.1 kg/m^2^), using a 128‐row helical CT scanner from Siemens, Germany. Subsequently, the CT scan data was imported into Mimics 21.0 software in Dicom format to establish a 3D model, which was then optimized using Geomagic Studio 2017 software.

**FIGURE 1:**
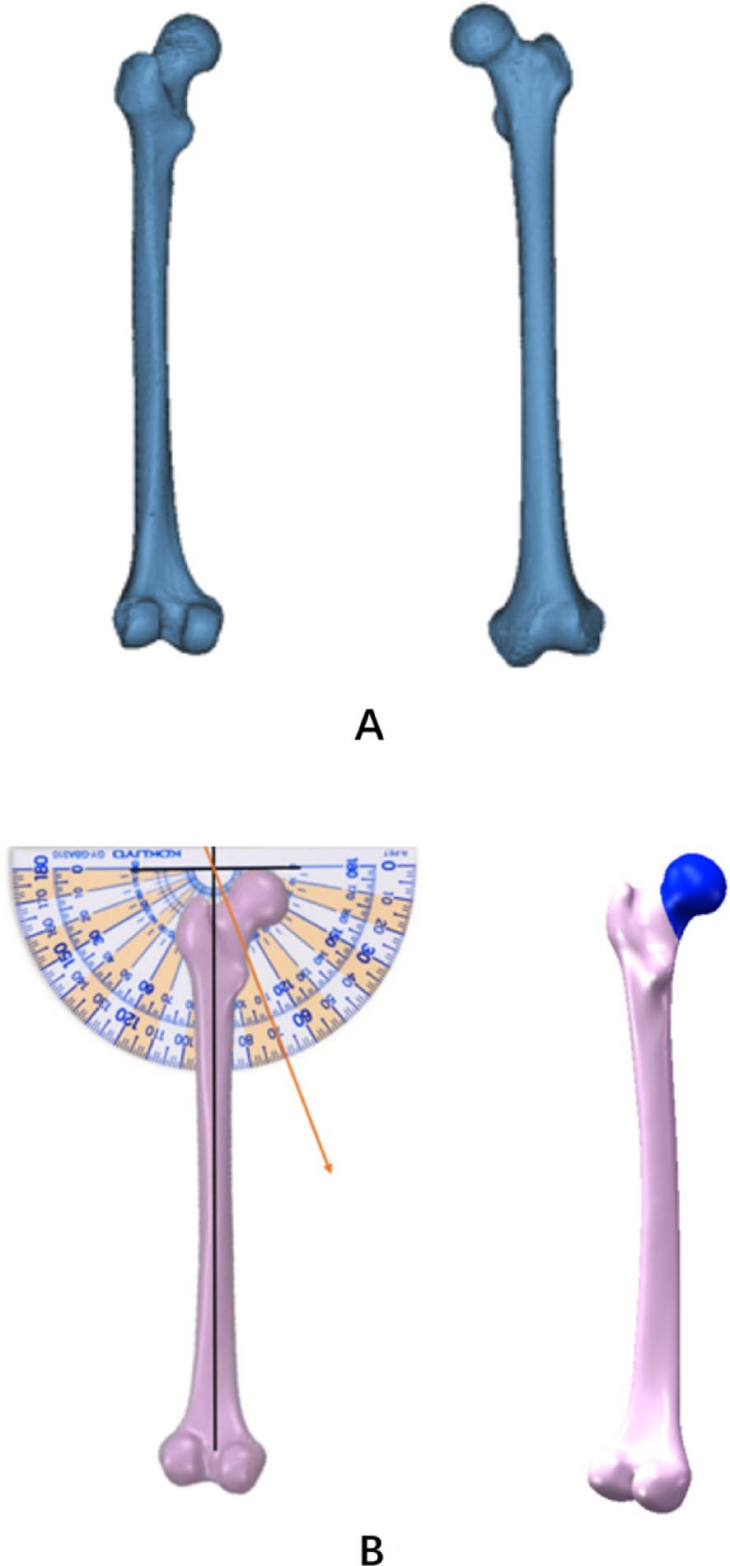
Three‐dimensional model of femur. (A) Anteroposterior and lateral views of the femoral model and (B) utilizing the modified Pauwels angle measurement method to determine the position of the fracture line and establish a fracture model.

**FIGURE 2:**
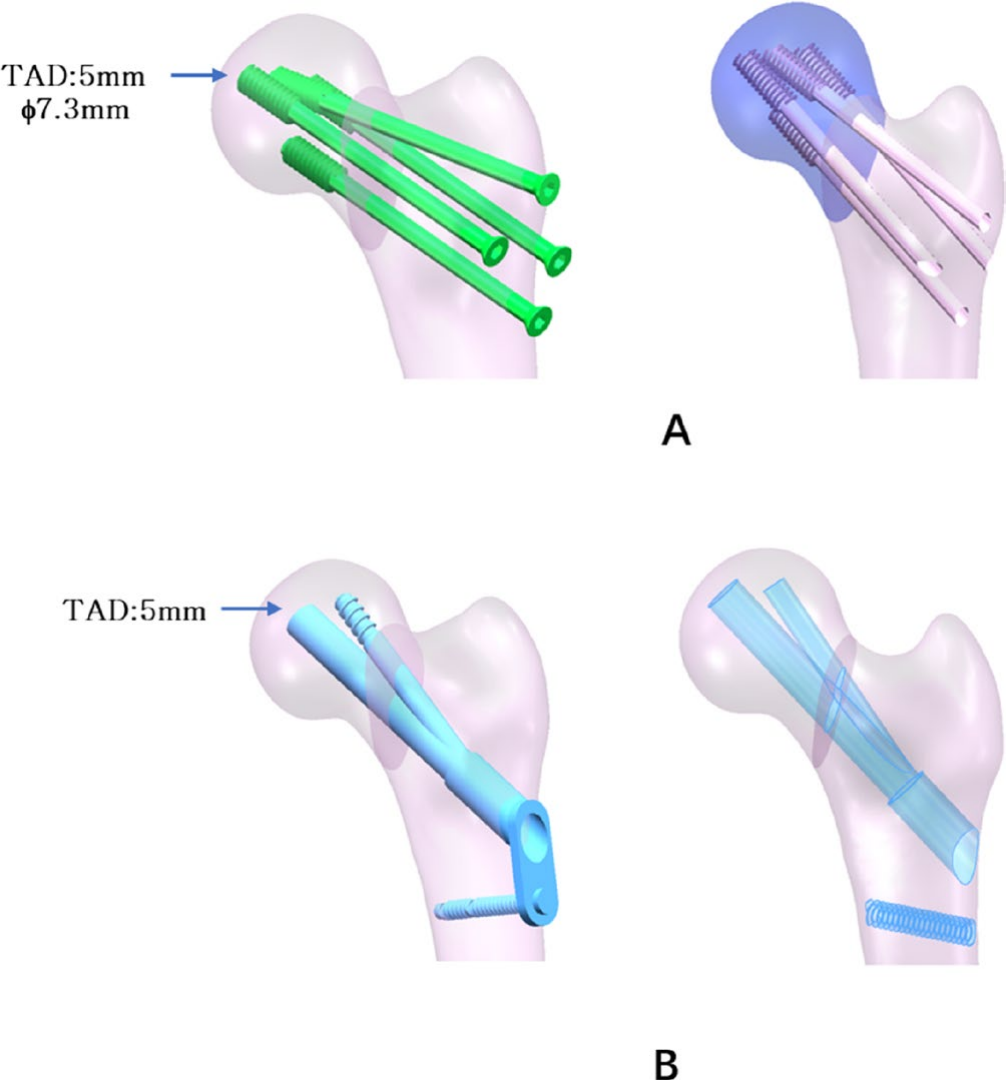
Establishment of fracture‐internal fixation model. (A) ICCS + TCS and (B) FNS.

Using SolidWorks 2020 software, a Pauwels III femoral neck fracture model was established based on the modified Pauwels classification [17], with an osteotomy angle of 70° (Figure 1). Based on the internal fixation parameters and surgical methods provided by the manufacturer, two internal fixation models were established (Figure 2).

##### 2.1.1.1 ICCS + Transversal Cannulated Screw (TCS)

The first screw should be inserted below the level of the lesser trochanter, close to the posterior inferior aspect of the femoral neck and adjacent to the femoral calcar. The second and third screws should be placed above the level of the lesser trochanter, respectively, at the anterior superior and posterior superior aspects of the femoral neck, close to the cortical bone of the femoral neck. All three cannulated compression screws should be parallel to the long axis of the femoral neck and form an inverted triangular structure. The tension band screw (TCS) is inserted perpendicular to the fracture line, located at the midline of the lateral wall of the femur and 15 mm below the apex of the greater trochanter. Each screw has a diameter of 7.3 mm, and the tip of the cannulated screw is 5 mm away from the subchondral bone of the femoral head.

##### 2.1.1.2 FNS

It consists of a dynamic rod, an anti‐rotation screw, and a one‐hole locking steel plate with a sleeve. The diameter of the power rod is 10 mm, the diameter of the hollow part is 2.6 mm, and the diameter of the anti‐rotation screw is 6.4 mm. The angle between the power rod and the locking steel plate is 130°, and the angle between the anti‐rotation screw and the power rod is 7.5°. A locking screw with a diameter of 5 mm is transversely locked on the femoral shaft to fix the steel plate and the femur. The power rod is located on the central axis of the femoral neck [18]. The tip of the power rod is located 5 mm below the cartilage of the femoral head.

#### 2.1.2 Material Properties and Boundary Conditions

Assuming that the femur and all implants exhibit linear elastic properties, the three‐dimensional experimental model was refined and meshed using Hypermesh 13.0 software to generate equal‐sized meshes with a size of 1 mm [19, 20]. Detailed counts of units and nodes are documented in Table 1.

**TABLE 1:** Number of nodes and elements of the model.

A: FNS		
Assembly part	Cortical bone	Cancellous bone
Cortical bone	40,765	208,884
Cancellous bone	32,612	167,107
FNS	85,387	19,417

B: ICCS + TCS		
Assembly part	Number of units	Number of nodes
Cortical bone	53,819	277,866
Cancellous bone	43,055	222,292
ICCS + TCS	16,421	58,992

Based on previous literature [21, 22], the contact between the femoral surface, fracture surface, and internal fixation‐cancellous bone is set as frictional contact. The friction coefficient for the fracture surface is set at 0.46, while that for the implant and cancellous bone is set at 0.3. The material properties of the model, such as Young's modulus and Poisson's ratio, are shown in Table 2.

**TABLE 2:** Material properties of femur and internal fixation.

Parameter	Titanium alloy (Ti‐6AL‐7Nb)	Cortical bone	Cancellous bone
Elasticity modulus (MPa)	110,000	16,800	840
Poisson ratio (v)	0.33	0.29	0.29

#### 2.1.3 FEA Stimulation and Main Outcome Measures

**FIGURE 3:**
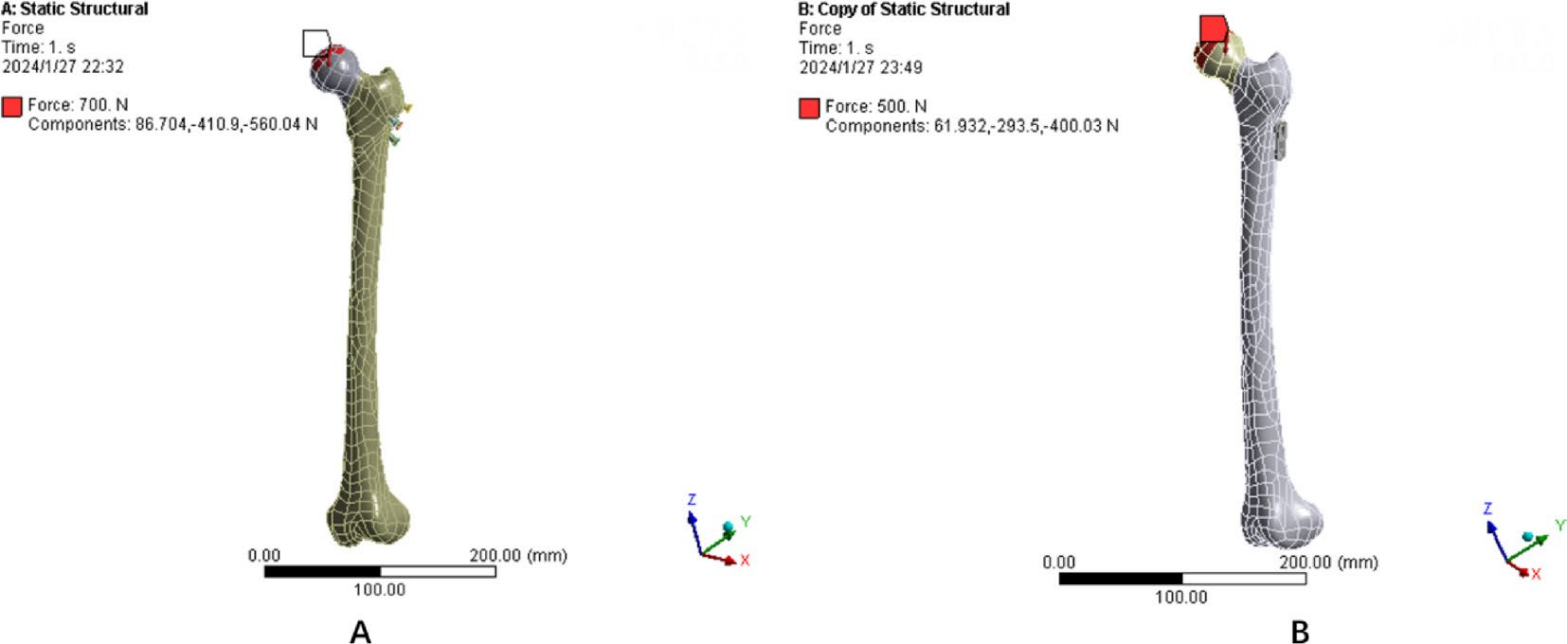
Diagram of the loading method. (A) ICCS + TCS and (B) FNS.

The femur is retracted by 10° [23, 24, 25, 26] in the coronal plane and tilted backward by 9° to simulate the weight‐bearing condition during single‐leg standing. Different gradients of load are applied to the center of the femoral head: 500, 1400, and 2100 N, simulating the stress conditions on the proximal femur under 0.7, 2, and 3 times the body weight of a 70 kg adult (Figure 3).

*Observation target:* (i) Displacement distribution and peak displacement of the femur and internal fixation; (ii) Von Mises stress distribution and peak stress of the femur and internal fixation; (iii) The boundary line between the load force line and the fracture plane is taken as the *Y* axis, the perpendicular line of the fracture plane is taken as the *X* axis, and the perpendicular line between the *X* axis and *Y* axis is taken as the *Z* axis; statistics on relative displacement in the *X*, *Y*, and *Z* directions.

### 2.2 Biomechanical Test

#### 2.2.1 Main Experimental Materials

**FIGURE 4:**
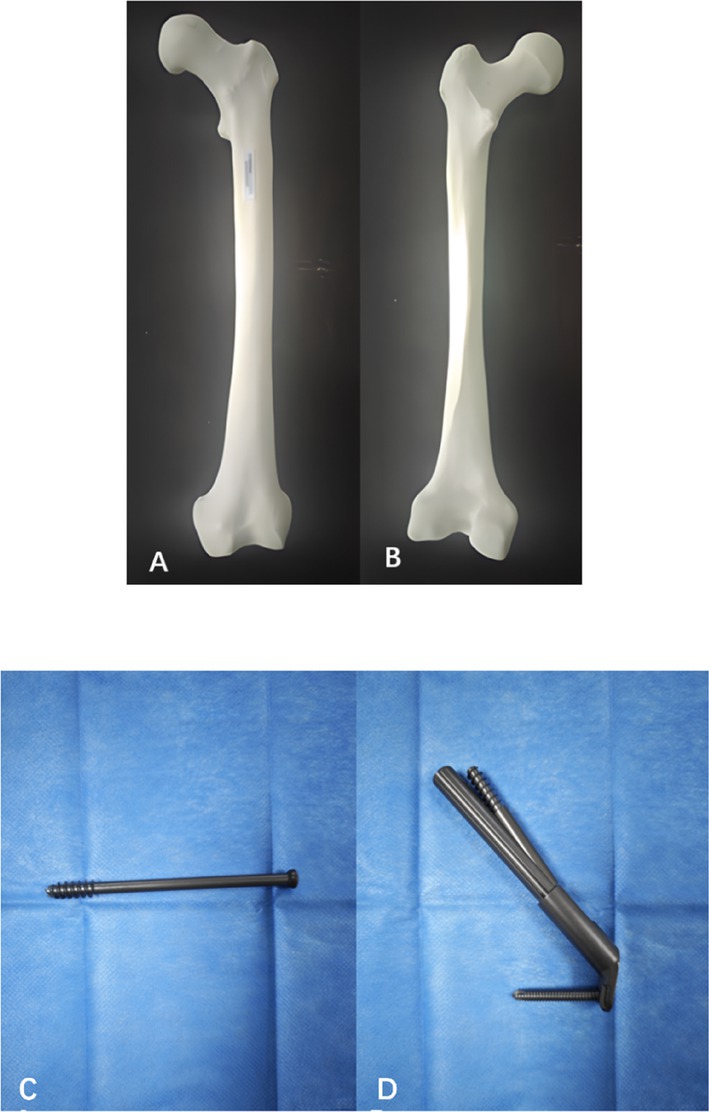
Experimental materials. (A and B) Artificial composite bone (Synbone LD2350, Left), (C) CCS, and (D) FNS.

The artificial composite femur (Synbone LD2350, Left, Switzerland) can represent most people and has been widely used in biomechanical experiments [27, 28]. Synbone LD2350 simulates the bone density of the femur in healthy adults aged 30–50 (approximately 0.8–1.0 g/cm^3^), which is consistent with the baseline of the clinical population in this study. *ϕ*7.3 mm hollow cancellous bone screw and matching tools (Sichuan Weisida Medical Equipment Co. Ltd., WSD2024012301); femoral neck minimally invasive fixation system and matching tools (Sichuan Weisida Medical Equipment Co. Ltd., WSD20240122) (Figure 4).

#### 2.2.2 Model Establishment and Experimental Scheme

**FIGURE 5:**
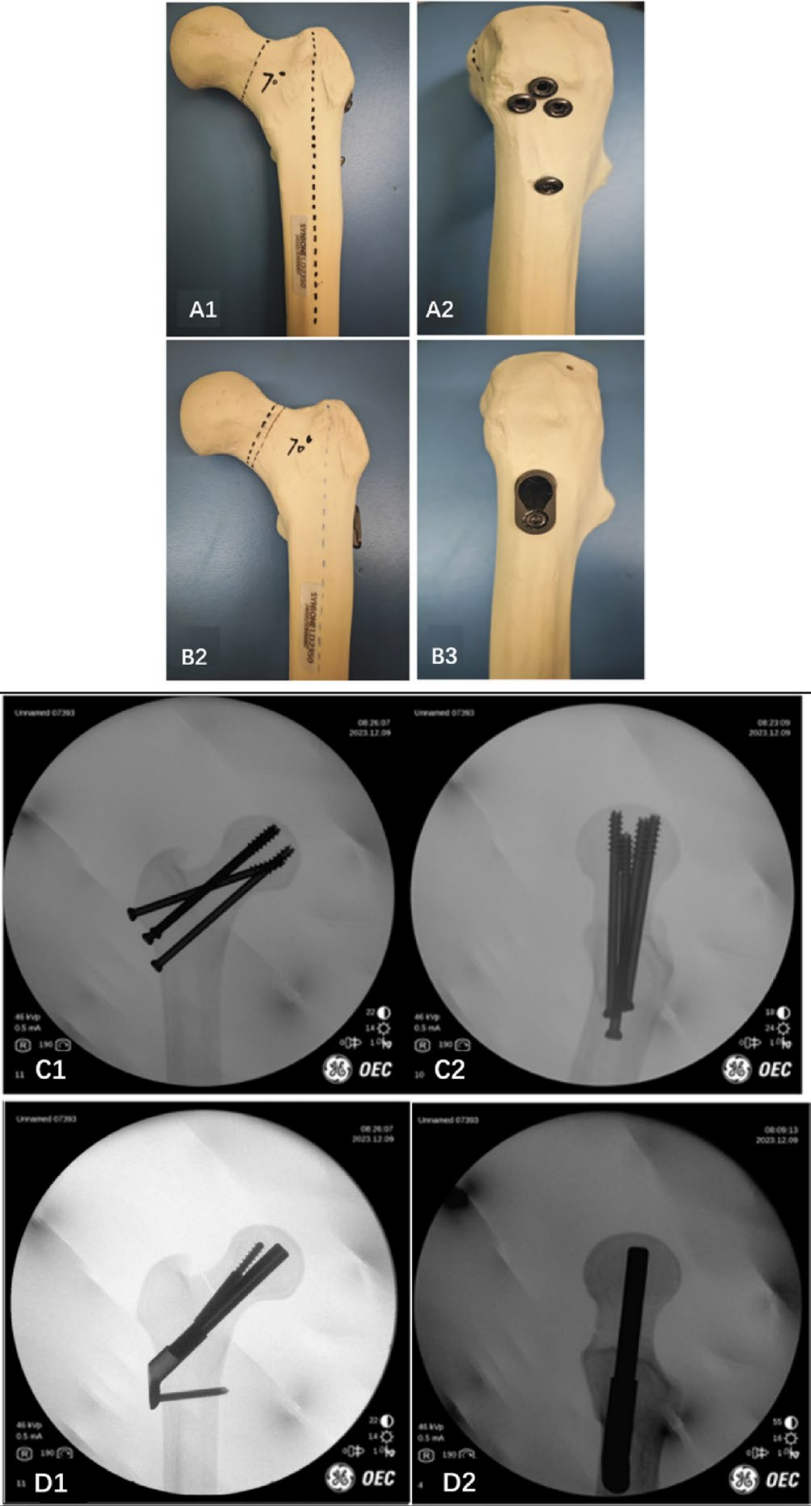
Establishment of internal fixation–fracture model. (A) ICCS + TCS, (B) FNS, (C) X‐ray after ICCS + TCS implantation, and (D) X‐ray after FNS implantation.

**FIGURE 6:**
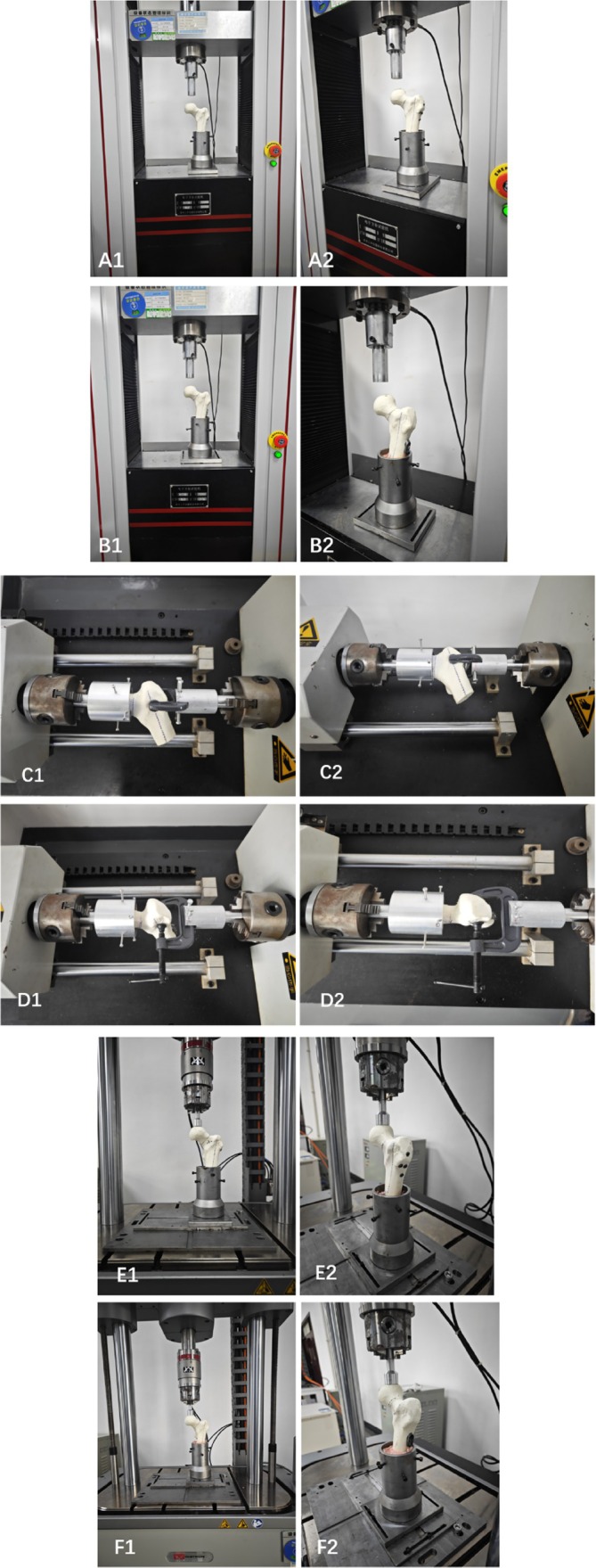
Mechanical experiments. (A and B) Static axial compression test (A1–2: ICCS + TCS, B1–2: FNS). (C and D) Static torsion test (C1–2: ICCS + TCS, D1–2: FNS). (E and F) Dynamic cyclic load test (E1–2: ICCS + TCS, F1–2: FNS).

Using an oscillating saw, a Pauwels III femoral neck fracture model was manually established, with the Pauwels angle positioned at 70°. Under C‐arm X‐ray fluoroscopy, ICCS + TCS and FNS were implanted using different methods, with the implantation position being the same as that of FEA. Each group consisted of 15 artificial composite bones (Figure 5). Then, these models were randomly divided into three groups for static axial compression tests, static torsion tests, and dynamic cyclic fatigue tests (Figure 6).

##### 2.2.2.1 Static Axial Compression Test

Artificial femoral specimens were cut at a distance of 20 cm from the proximal end of the femur, and the femur was retracted by 10° in the coronal plane and tilted backward by 9° in the sagittal plane to simulate the hip joint stress environment during single‐leg standing [29]. The mechanical indenter uses a concave indenter similar to the acetabulum. Before the experiment begins, a preload of 100 N is applied to eliminate potential movement space between the internal fixation and the bone [30]. The loading speed is set at 6 mm/min, and the termination conditions are set at 2100 N, with a relative displacement between fracture blocks exceeding 5 mm, internal fixation failure, and femoral neck re‐fracture [31, 32].

##### 2.2.2.2 Static Torsion Test

Apply a torsional load starting from 0 N·m, and rotate the femoral head clockwise at a speed of 6°/min (0.06°/s) until it reaches 10°, or until the model exhibits failure such as fracture or screw backout [33].

##### 2.2.2.3 Dynamic Cyclic Loading Experiment

Using the same experimental conditions as the static axial compression experiment, the frequency is set to 2 Hz, and the total number of cycles is set to 10,000 [34]. When a 70‐kg person walks with both legs, the hip joint bears a stress load of 700 N [35], and the number of cycles is approximately equivalent to the number of steps taken during the 4–6 weeks of fracture healing [36].

#### 2.2.3 Observation Indicators

The load‐deformation curve is obtained from the static compression experiment, and the axial stiffness is determined by the slope of the curve. The torque‐angle curve is plotted through the torsion experiment, with the torsional stiffness being the slope of the curve. The torque at different angles is recorded. The overall deformation generated by dynamic cycling is evaluated by recording the displacement through the software system built into the mechanical testing machine, reflecting the load‐bearing deformation during the fracture healing period.

### 2.3 Clinical Study

#### 2.3.1 Subjects and Methods

A retrospective analysis was conducted on the medical records of 21 patients treated with ICCS + TCS for fresh Pauwels type III adult femoral neck fractures admitted to the Department of Orthopedics and Joint Surgery, Affiliated Hospital of Southwest Medical University, from January 2023 to December 2024. The internal fixation material used in the study was a *ϕ*7.3 mm hollow cancellous screw (Sichuan Weisida Medical Instrument Co. Ltd.), made of titanium alloy with excellent biocompatibility. This study was approved by the Ethics Committee of the Affiliated Hospital of Southwest Medical University (Ethics Approval Number: KY2023318). All patients provided informed consent for the treatment plan and voluntarily participated in this study.

#### 2.3.2 Experimental Standards

*Inclusion criteria:* (1) Imaging diagnosis of Pauwels type III femoral neck fracture (The angle between the femoral neck fracture line and the horizontal line is ≥ 50°, representing a trans‐cervical femoral neck fracture, Garden II, III, IV type); (2) Complete medical records; (3) Age < 65 years; (4) Fixation method of ICCS + TCS; and (5) follow‐up duration > 6 months.

*Exclusion criteria:* Open fractures or pathological fractures.

#### 2.3.3 Surgical Method

All surgeries were performed by associate chief physicians or higher‐level doctors from the same treatment team, initially utilizing the Leadbetter flexion–extension reduction method [37]. Patients were placed in a supine position on an orthopedic traction table, and under C‐arm fluoroscopy, reduction was achieved through abduction traction followed by adduction and internal rotation. If closed reduction fails to achieve satisfactory results after being repeated 3 times, it is necessary to combine percutaneous pinning or adopt the Smith–Petersen approach for direct vision reduction. Once the reduction standard is met, which means both the frontal and lateral radiographs show a double “S” curve, maintain traction.

After achieving satisfactory reduction of the fracture, a transverse guide wire perpendicular to the fracture line is first placed under C‐arm fluoroscopy. Then, three guide pins were implanted according to the traditional ICCS method. Under the frontal perspective, the three guide pins were parallel; under the lateral perspective, the three guide pins were distributed in a dispersed manner, and they were placed as close to the cortex as possible, with the tips all located 5 mm below the femoral head cartilage. First, measure the depth of the guide wire perpendicular to the fracture line, ream the medullary canal, and screw in the screw. Then, proceed with the rest of the guide wires in turn. After flushing the incision, suture it and apply a dressing for bandaging. Antibiotics were used once after operation to prevent infection. Patients were instructed to perform ankle pump exercises and isometric contractions of the quadriceps muscle, combined with low molecular weight heparin, to prevent the formation of deep venous thrombosis in the lower limbs.

Rehabilitation exercises and postoperative weight‐bearing time are conducted in accordance with relevant literature and guidelines [38, 39].

#### 2.3.4 Result Evaluation

(1) Record the patient's perioperative condition. (2) Evaluating fracture reduction through the Garden index: I, with a normal position of 160° in the frontal plane and 180° in the lateral plane; II, with a normal position of 155° in the frontal plane and 180° in the lateral plane; III, with a normal position of < 150° in the frontal plane or > 180°in the lateral plane; IV, with a normal position of 150° in the frontal plane and > 180° in the lateral plane. (3) The hip joint function of the affected limb is assessed using the Harris score (out of 100, with higher scores indicating better function), hip joint flexion–extension range of motion (ROM), and hip joint internal–external rotation ROM. (4) Evaluate fracture healing by assessing radiographic findings, fracture healing time, and measuring the distance of femoral neck shortening using the Mose concentricity method [40].

### 2.4 Statistical Methods

SPSS27.0 statistical software was used for analysis. Data were expressed as $\bar{x}\pm s$, and data that conformed to normal distribution and homogeneity of variance were analyzed using one‐way analysis of variance and independent sample *t*‐test. LSD *t*‐test was used for inter‐group comparison Sample data that do not conform to the normal distribution are represented by the median (interquartile range), and Kruskal–Wallis *H* tests are used for overall comparisons and pairwise comparisons between sample groups. *p* < 0.05 indicates a statistically significant difference.

## 3 Result

### 3.1 The Results of Finite Element Analysis

#### 3.1.1 Peak Stress and Relative Displacement of Femur

**FIGURE 7:**
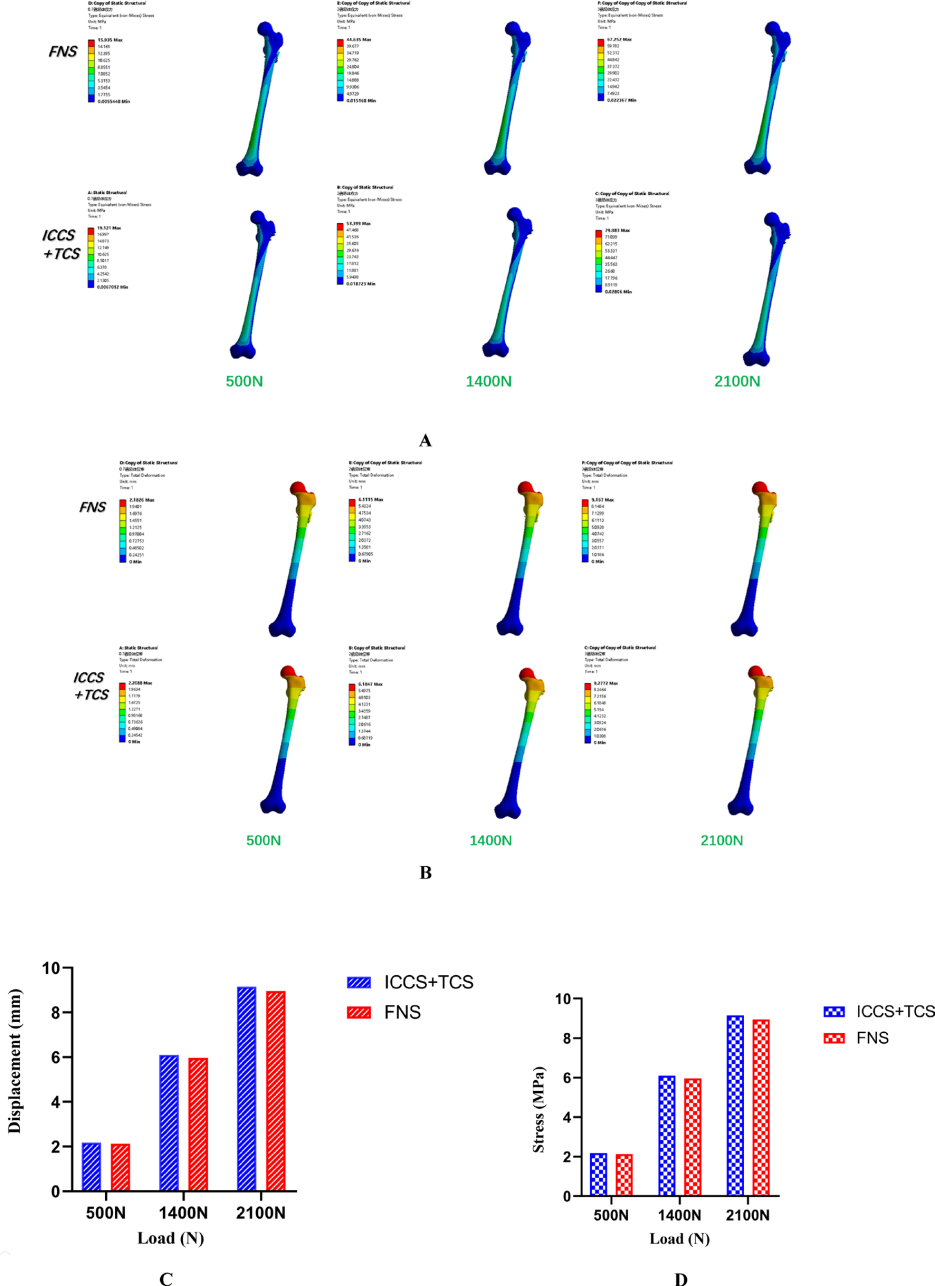
Finite element analysis results of femur. (A) Stress distribution under different loads on the femur. (B) Displacement distribution under different loads on the femur. (C) Relative displacement of femur (mm). (D) Femur stress distribution (MPa).

As shown in Figure 7, both sets of stresses are concentrated at the fracture end of the femoral calcar and the medial side of the femoral shaft. The maximum displacement occurs at the proximal load‐bearing area of the femoral head, with displacement gradually decreasing from proximal to distal. The femoral displacement and stress under 500, 1400, and 2100 N for ICCS + TCS and FNS are shown in Table 3. There is no significant difference in femoral displacement between ICCS + FNS and FNS (*p* > 0.05, there was no significant difference in statistics), but the femoral stress is greater for ICCS + FNS than for FNS (*p* < 0.05, there was a significant difference in statistics).

**TABLE 3:** Finite element analysis results of femur.

Parameter	Load force	ICCS + TCS	FNS	*p*
Relative displacement of femur (mm)	500 N	2.21	2.18	*p* > 0.05
	1400 N	6.18	6.11	*p* > 0.05
	2100 N	9.28	9.17	*p* > 0.05
Femurstress distribution (MPa)	500 N	19.12	15.94	*p* > 0.05
	1400 N	53.34	44.64	*p* > 0.05
	2100 N	79.98	67.25	*p* > 0.05

#### 3.1.2 Peak Values of Internal Fixation Stress and Relative Displacement

**FIGURE 8:**
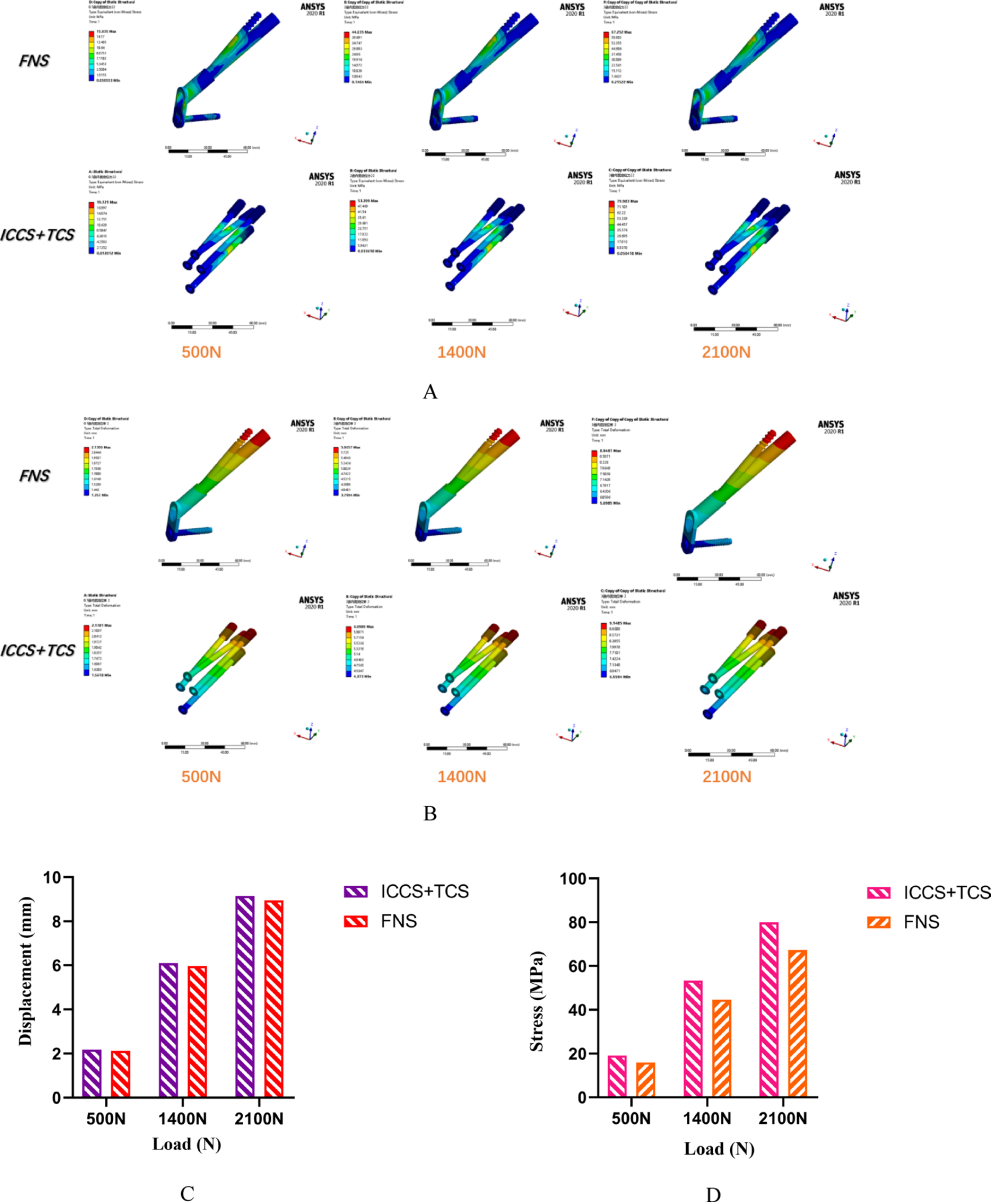
Finite element analysis results of internal fixation. (A) Stress distribution under different loads on the internal fixation. (B) Displacement distribution under different loads on the internal fixation. (C) Relative displacement of internal fixation (mm). (D) Internal fixation stress distribution (MPa).

As shown in Figure 8, the displacement peaks of both groups are concentrated at the tip of the screw. The displacement peaks of ICCS + TCS are mainly concentrated at the tips of the upper three hollow screws. Both groups exhibited stress concentration at the fracture ends. In the ICCS + TCS group, the maximum stress was located at the screw near the femoral calcar, while in the FNS group, the maximum stress was found at the antirotation screw. Additionally, some stress of FNS was transferred to the locking plate. Specific results are shown in Table 4. There was no significant difference in displacement between ICCS + FNS and FNS (*p* > 0.05), but the stress of ICCS + FNS was greater than that of FNS (*p* < 0.05). Compared to FNS, the CCS located below is closer to the Calcar femorale, where stress is more concentrated, and the diameter of the CCS is significantly smaller than that of the FNS dynamic rod, making it less resistant to ultimate load. Therefore, the possibility of screw fracture in ICCS + TCS is greater compared to FNS.

**TABLE 4:** Finite element analysis results of internal fixation.

Parameter	Load force	ICCS + TCS	FNS	*p*
Relative displacement of internal fixation (mm)	500 N	2.18	2.13	*p* > 0.05
	1400 N	6.10	5.97	*p* > 0.05
	2100 N	9.15	8.95	*p* > 0.05
Internal fixation stress distribution (MPa)	500 N	19.12	15.94	*p* < 0.05
	1400 N	53.40	44.64	*p* < 0.05
	2100 N	79.98	67.25	*p* < 0.05

#### 3.1.3 Relative Displacement of Fracture Section

**FIGURE 9:**
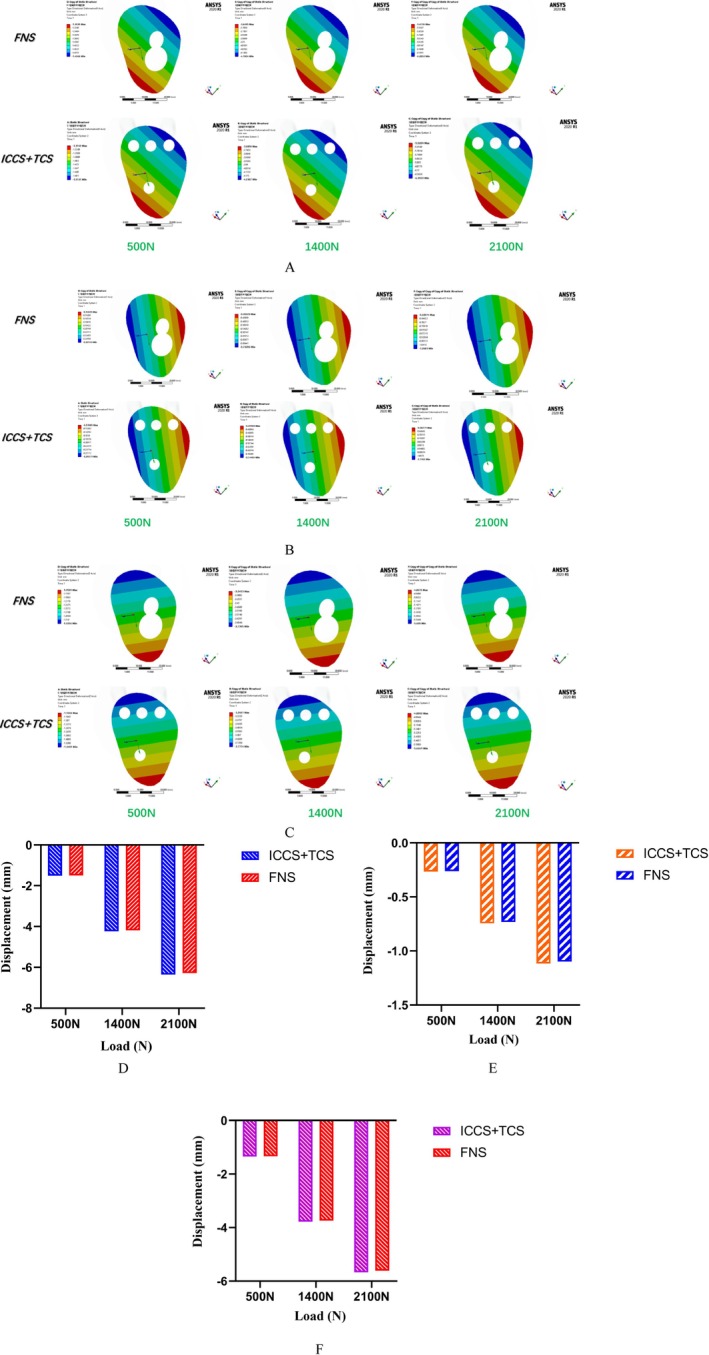
Relative displacement of fracture section. (A and D) Relative displacement along the *X*‐axis. (B and E) Relative displacement along the *Y*‐axis. (C and F) Relative displacement along the *Z*‐axis.

As shown in Figure 9, the relative displacement of the *X*, *Y*, and *Z* axes represents the ability to resist fracture varus deformation, vertical shear, and torsional deformation. The *X*‐axis displacement of the two groups is concentrated on the upper posterior side of the fracture section, the *Y*‐axis on the anterior side of the fracture section, and the *Z*‐axis on the upper anterior side of the fracture section. There is no significant difference between the relative displacement of the *X*, *Y*, and *Z* axes in ICCS + TCS and that in FNS (*p* > 0.05) (Table 5).

**TABLE 5:** Relative displacement of fracture section.

Parameter	Load force	ICCS + TCS	FNS
Relative displacement of *X*‐axis (mm)	500 N	−1.51	−1.50
	1400 N	−4.24	−4.19
	2100 N	−6.36	−6.29
Relative displacement of *Y*‐axis (mm)	500 N	−0.27	−0.26
	1400 N	−0.74	−0.73
	2100 N	−1.12	−1.10
Relative displacement of *Z*‐axis (mm)	500 N	−1.35	−1.34
	1400 N	−3.78	−3.74
	2100 N	−5.67	−5.61

### 3.2 The Results of Biomechanical Test

#### 3.2.1 Static Axial Compression Experiment

**FIGURE 10:**
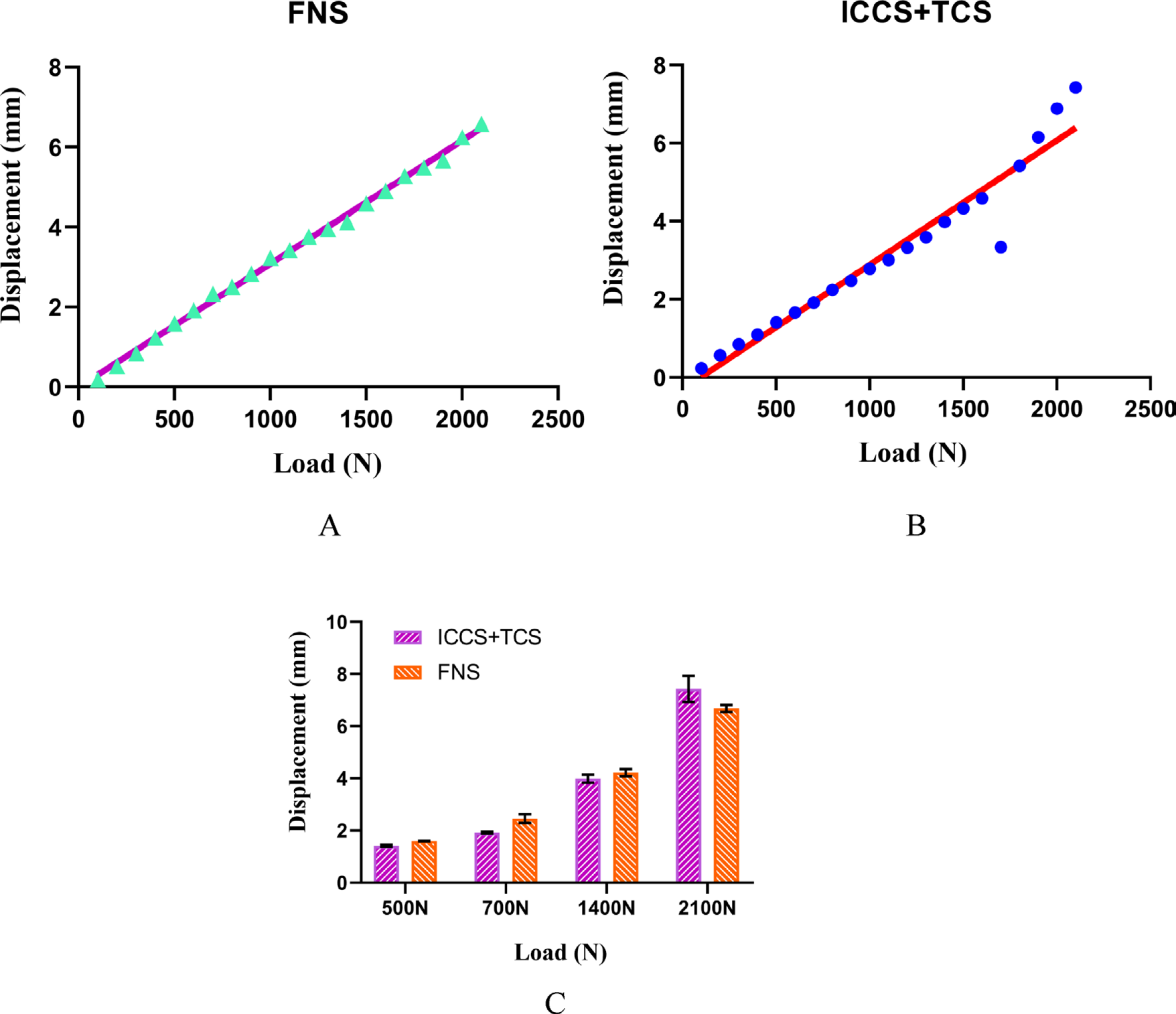
Static axial compression experiment. (A) The axial stiffness of FNS (mm). (B) The axial stiffness of ICCS + TCS (mm). (C) Relative displacement under different loads (mm).

In the nondestructive continuous load static compression test, no model experienced internal fixation failure, fracture, or femoral fracture. As shown in Figure 10, under continuous static axial compression conditions, the axial stiffness of ICCS + TCS is 317.06 ± 33.21 N/mm, which is lower than that of FNS, which is 324.36 ± 8.20 N/mm (*p* < 0.05, the difference was statistically significant).

#### 3.2.2 Static Torsion Test

**FIGURE 11:**
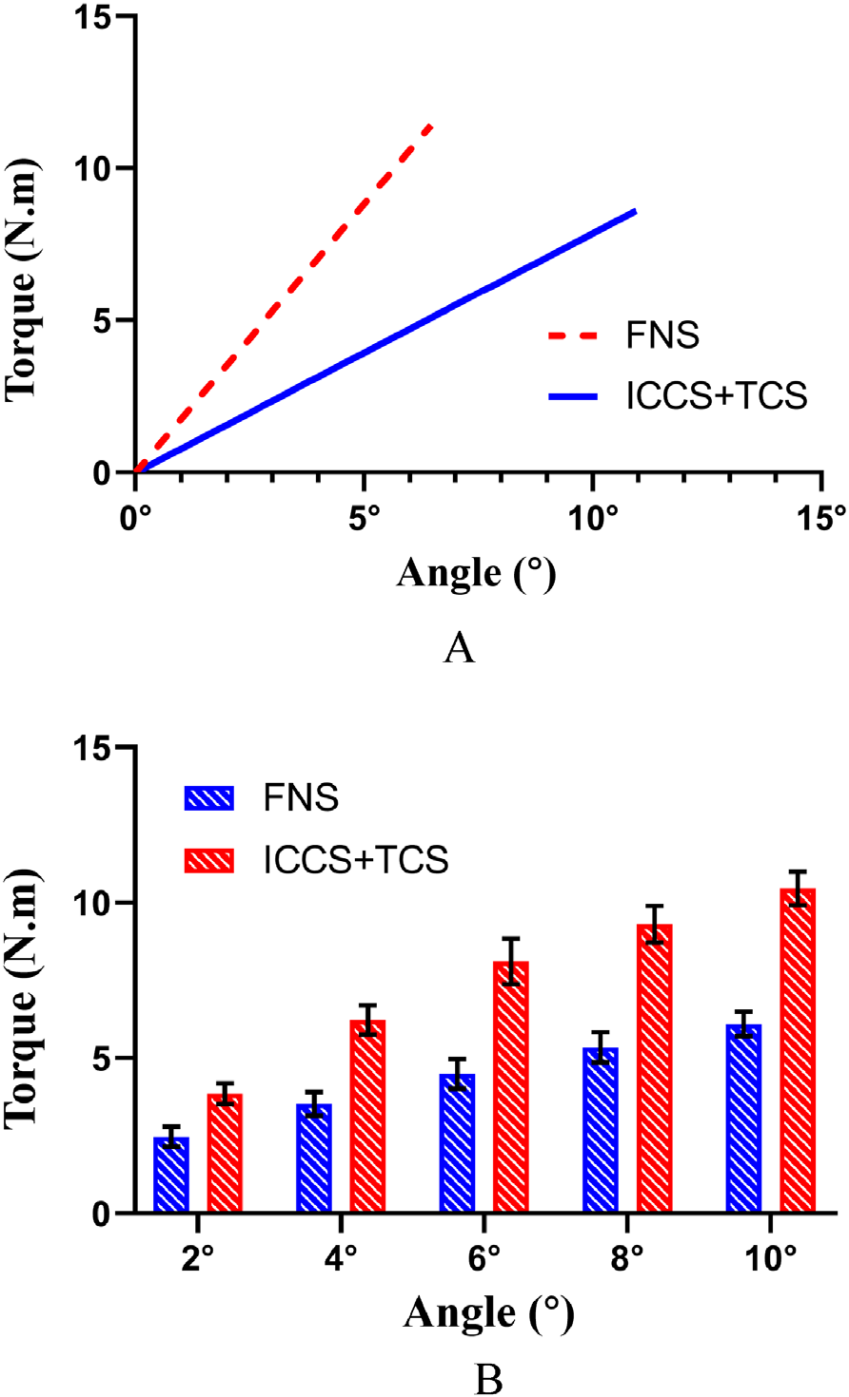
Static torsion test. (A) Axial stiffness of the two sets of models (N m). (B) Torque of two sets of models under different torsion angles (N m).

In the continuous torsion test, no model experienced internal fixation failure, fracture, or femoral fracture. As shown in Figure 11A, the torsional stiffness of ICCS + TCS is 1.05 ± 0.01 N·m (°), which is significantly higher than that of FNS, which is 0.55 ± 0.05 N·m (°) (*p* < 0.05, The difference was statistically significant). At torsion angles of 2°, 4°, 6°, 8°, and 10°, the torque of ICCS + TCS is greater than that of FNS (*p* < 0.05, The difference was statistically significant) (Figure 11B).

#### 3.2.3 Dynamic Cyclic Loading Test

**FIGURE 12:**
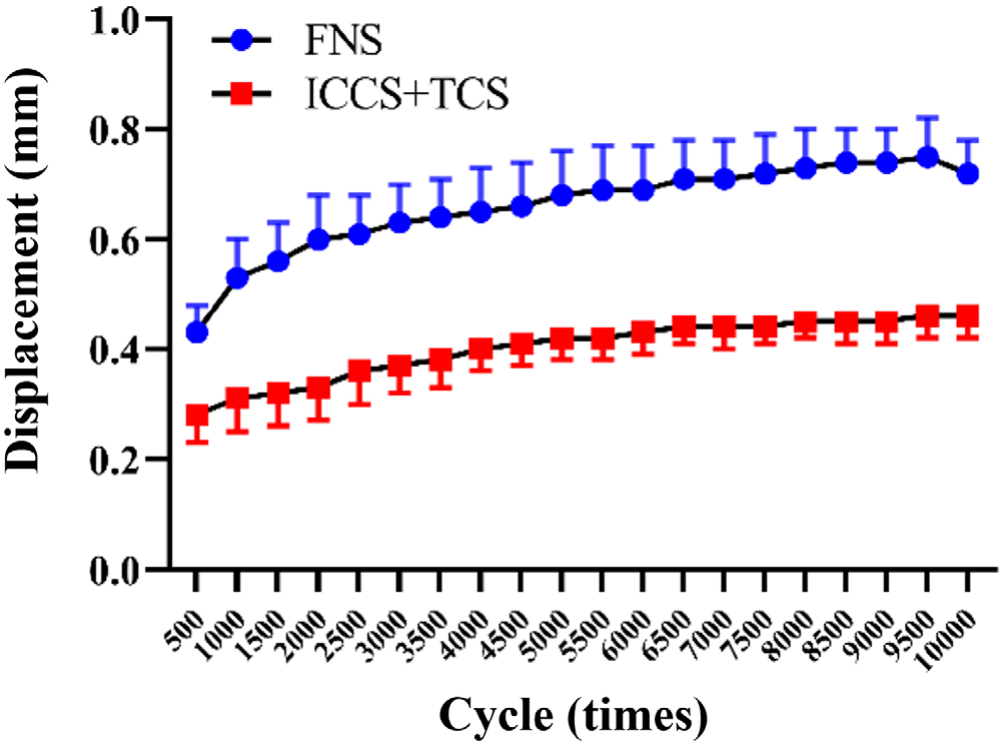
Dynamic cyclic loading test.

In the cyclic loading experiment, no model experienced internal fixation failure, fracture, or femoral fracture. As shown in Figure 12, the overall deformation of ICCS + TCS is 0.46 ± 0.04 mm, significantly lower than that of FNS, which is 0.76 ± 0.06 mm (*p* < 0.05, The difference was statistically significant).

### 3.3 Clinical Retrospective Study

#### 3.3.1 Perioperative Period

Among the 21 patients with ICCS + TCS, 14 were male and seven were female, with an average age of 48.71 ± 10.27 years. There were seven cases of car accident injuries, nine cases of high fall injuries, and five cases of fall‐related injuries. The preparation time from injury to surgery was 0.95 ± 0.69 days, and all fractures were of Pauwels type III femoral neck fractures.

All patients successfully completed the surgery. Seven patients underwent successful closed reduction, 13 patients underwent successful reduction through percutaneous needle prying, and two patients underwent successful reduction under direct vision through the Smith‐Petersen approach.

None of the 21 patients experienced infection, allergic reaction, immune response, or rejection around the internal fixation. The perioperative data are shown in Table 6.

**TABLE 6:** Perioperative data.

Outcome measures	Parameter
Age ($\bar{x}$ ± s, years)	48.71 ± 10.27
Gender (male/female, *n*)	14/7
Injury mechanism (car accident injury/high fall injury/fall injury, *n*)	7/9/5
Operative time ($\bar{x}$ ± s, min)	91.48 ± 11.89
Intraoperative blood loss ($\bar{x}$ ± s, mL)	14.62 ± 4.47
Intraoperative fluoroscopy times ($\bar{x}$ ± s, times)	35.90 ± 7.75
Total length of incision ($\bar{x}$ ± s, cm)	3.63 ± 0.50
Surgical methods (closed reduction/percutaneous prying/open reduction, *n*)	7/12/2

#### 3.3.2 Postoperative Follow‐Up

**FIGURE 13:**
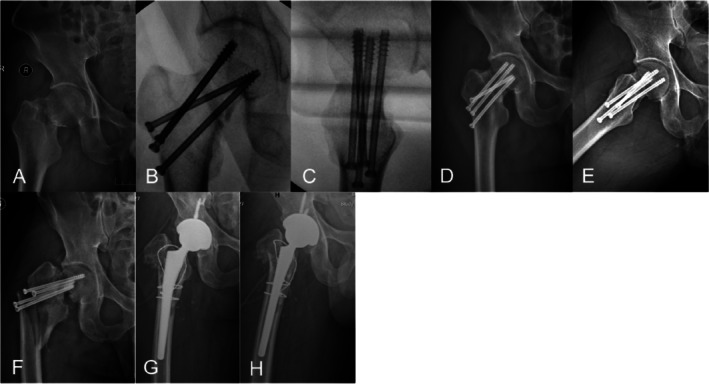
Patient, male, 41 years old, fall‐related injury; preoperative X‐ray of the first operation (A); X‐ray after the first operation (B and C); X‐ray after 1 month (D and E); X‐ray after the second injury (F); X‐ray at 5 months after the second operation (G and H).

**FIGURE 14:**
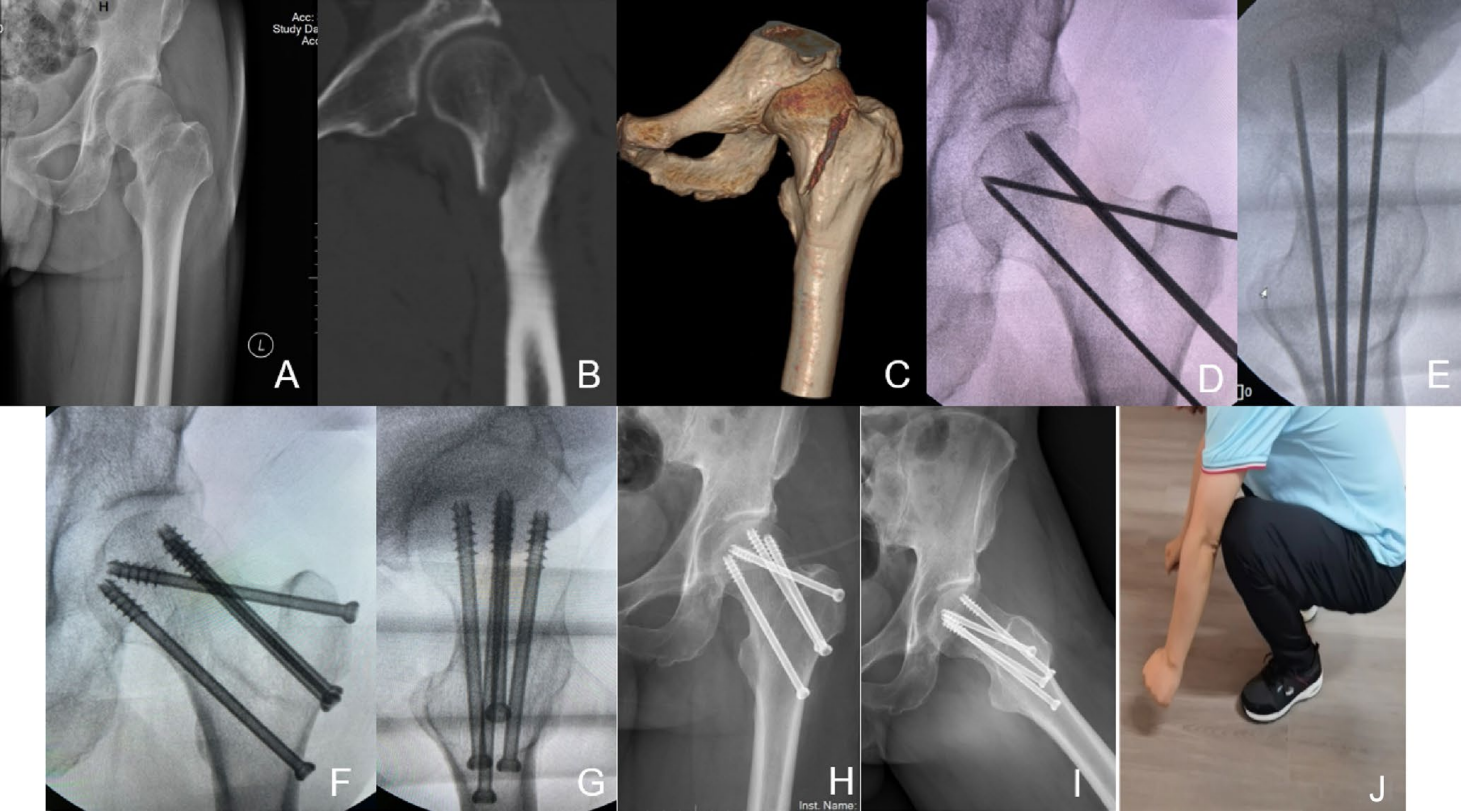
Patient, male, 47 years old, with left femoral neck fracture. (A–C) Preoperative X‐ray and CT scans revealed a Pauwels III femoral neck fracture on the left side. (D–G) Postoperative lateral X‐ray demonstrates good reduction of the fracture, and the placement of the hollow screw is satisfactory. (H–J) The last follow‐up X‐ray and the patient's hip joint functional position.

**FIGURE 15:**
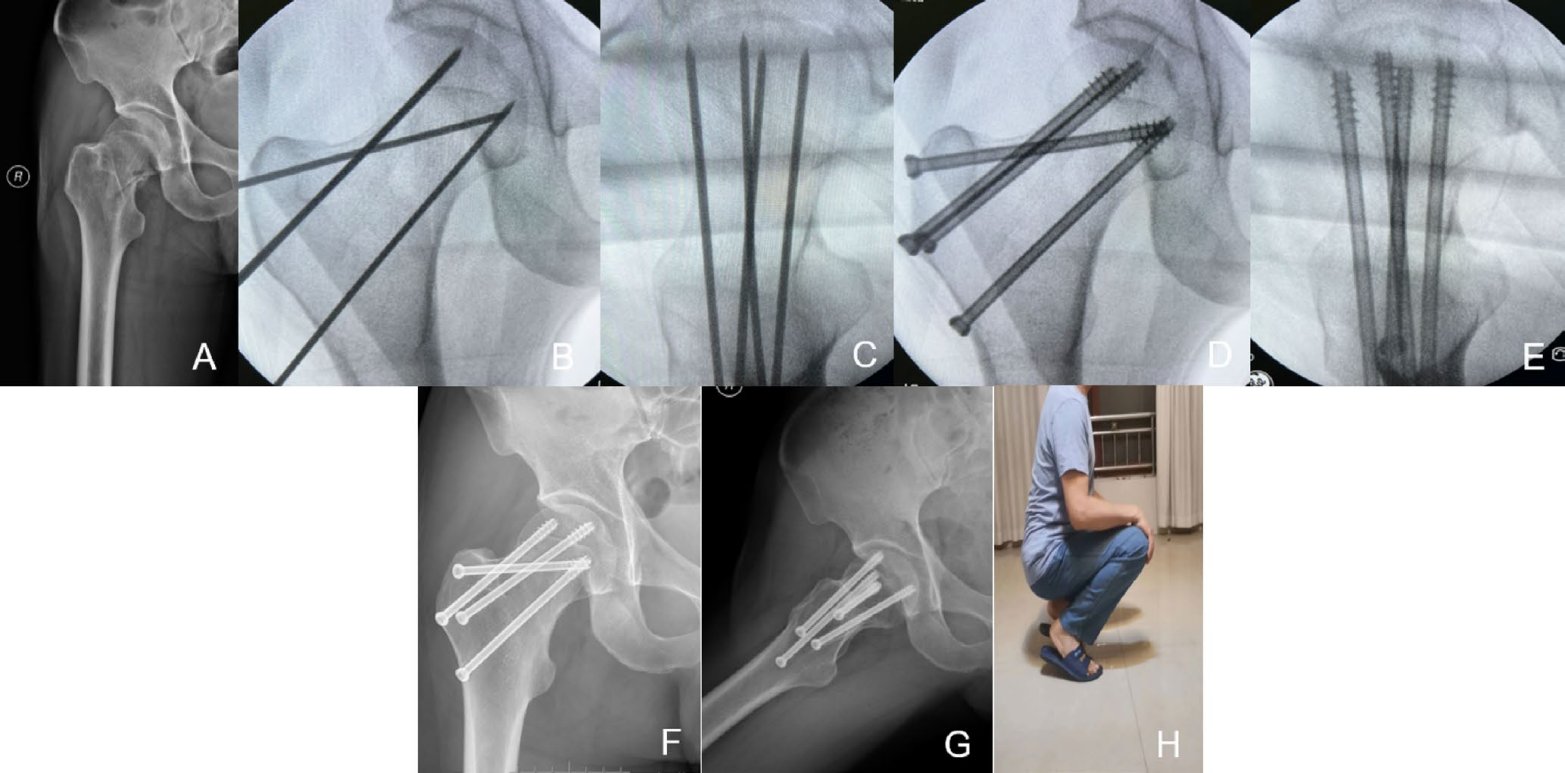
Patient, male, 52 years old, with right femoral neck fracture. (A) Preoperative X‐ray revealed a Pauwels III femoral neck fracture on the right side. (B–E) Postoperative lateral X‐ray demonstrates good reduction of the fracture, and the placement of the hollow screw is satisfactory. (F–H) The last follow‐up X‐ray and the patient's hip joint functional position.

Except for one patient who underwent joint replacement surgery due to a subtrochanteric fracture of the femur caused by a car accident 1 month after surgery (patient, male, 41 years old, preoperative examination did not indicate osteoporosis, and the internal fixation device did not loosen or break, as shown in Figure 13), due to high‐energy violence, the patient suffered from ipsilateral intertrochanteric fracture of the femur, which subsequently led to failure of internal fixation [41]. All 20 patients were followed up for a period ranging from 8 to 25 months, with an average of 16.35 ± 4.51 months. Harris score, hip flexion‐extension ROM, and internal–external rotation ROM all showed significant increases over time (*p* < 0.05). The difference was statistically significant (Table 7). Typical cases are shown in Figures 14 and 15.

**TABLE 7:** Postoperative follow‐up data.

Outcome measures	Parameter
Follow‐up (months)	16.35 ± 4.51
Extension‐flexion ROM 3 months postoperatively (°)	110.05 ± 6.40
Extension‐flexion ROM at the last follow‐up (°)	129.35 ± 5.98
Internal rotation‐external rotation ROM 3 months postsurgery (°)	28.0 ± 5.16
Internal rotation‐external rotation ROM at the last follow‐up (°)	44.15 ± 4.79
Harris score 3 months after surgery	65.10 ± 10.28
Harris score at the last follow‐up	88.10 ± 3.94
Time for full weight‐bearing (weeks)	25.65 ± 3.17
Garden alignment index (I/II/III/IV, *n*)	15/4/1/0
Shortening length of femoral head neck immediately after surgery ($\bar{x}$ ± s, mm)	0.89 ± 0.41
Shortening length of femoral head neck immediately at the last follow‐up ($\bar{x}$ ± s, mm)	3.87 ± 0.44
Fracture healing time (weeks)	23.95 ± 5.13

## 4 Discussion

Based on the results of this study, we can conclude that ICCS + TCS exhibits biomechanical trends compared to FNS; meanwhile, it has stronger torsional resistance and load‐bearing deformations. Simultaneously, ICCS + TCS has demonstrated favorable outcomes in clinical treatments. This study offers a novel approach for the treatment of P3FNFs.

Femoral neck fractures currently constitute a significant type of hip fractures in China [2]. Complications arising from fractures, such as hip joint dysfunction, femoral head necrosis, and nonunion, have caused immense suffering and financial burden to patients and their families, and have delayed social development. For young and middle‐aged patients, due to their longer life expectancy and high functional requirements of the hip joint, hip‐preserving treatment is generally preferred. P3FNFs represent a type of vertical unstable femoral neck fracture, characterized by high shear and torsional forces at the fracture site, which can easily lead to implant breakage, screw backout, femoral head varus and rotation around neck, resulting in related complications in patients [42]. Therefore, the key factors in the treatment of Pauwels type III femoral neck fractures are anatomical reduction and rigid fixation.

There is controversy over the optimal treatment method for Pauwels type III femoral neck fractures. CCS has advantages such as minimally invasive implantation, compression of the fracture ends, and being economical and practical, making it widely promoted and used in China [43]. The screw configuration chosen for the CCS placement scheme is one of the main factors affecting the biomechanical performance of its fixation of the femoral neck. In China, the most common method is the inverted triangular screw placement parallel to the long axis of the femoral neck. However, existing literature has reported that it cannot effectively reconstruct and maintain the biomechanical environment of the femoral neck when treating vertical femoral neck fractures [44]. Some scholars have proposed improved screw placement methods such as strong oblique screw placement, cross screw placement, F‐configuration, and other biplanar bi‐support methods for the fixation of Pauwel III femoral neck fractures [45, 46, 47, 48]. However, multiple studies have reported that regardless of the chosen spatial structure distribution, three hollow screws cannot provide strong fixation for Pauwels type III femoral neck fractures [49, 50, 51]. A biomechanical analysis conducted by Gümüştaş [52] showed that a combination of three parallel inverted triangular hollow screws and 1 transverse hollow screw directed toward the femoral calcar can provide excellent stability in the fixation of unstable femoral neck fractures. Currently, other studies [53, 54, 55, 56] have shown that combining an additional hollow screw with ICCS can effectively enhance the biomechanical efficiency of femoral neck fixation.

### 4.1 Biomechanics Validation

FNS is currently one of the mainstream solutions for treating vertically unstable femoral neck fractures, demonstrating excellent mechanical properties and therapeutic effects [57, 58, 59]. Through finite element analysis, this study predicts that ICCS + TCS can exhibit biomechanical properties that are very similar to those of FNS. Due to the coarser dynamic rod of FNS and its ability to transmit stress to the femoral shaft through the locking plate, it can reduce the peak stress [60]. Therefore, FNS has a more advantageous position in resisting vertical shear forces.

Based on the configuration of the rhombic hollow screw, this article proposes to replace the fourth hollow screw by inserting it into the lateral wall of the femur in a direction perpendicular to the fracture line. Currently, there are relatively few relevant literature reports on this method, and they are mainly focused on theoretical directions. Simultaneously, we conducted finite element analysis, in vitro biomechanical testing, and clinical research to compare the new internal fixation device FNS, thereby verifying the effectiveness of ICCS + TCS in treating Pauwels type III femoral neck fractures.

Through finite element analysis, this study predicts that ICCS + TCS can exhibit biomechanical properties that are very similar to those of FNS. Due to the coarser dynamic rod of FNS and its ability to transmit stress to the femoral shaft through the locking plate, it can reduce the peak stress. This is consistent with the subsequent in vitro biomechanical prediction trend results, indicating that FNS can withstand higher vertical shear forces and reduce the risk of internal fixation fracture during repeated vertical downward stress.

Through in vitro biomechanical experiments, we further explored the biomechanical properties of ICCS + TCS. The results indicated that it exhibited significant advantages over FNS in terms of resistance to torsion and fatigue deformation. This may be attributed to TCS's ability to better compress the fractured ends, increase the friction between the fracture blocks, and simultaneously, the four screws can form a three‐dimensional space and effectively occupy the internal space of the femoral head, stably holding the femoral head, enhancing anti‐rotation ability, and effectively preventing the femoral head from rotating around the axis, thus providing a strong fixation effect [61]. In FNS, the dynamic rod and locking plate form a 130° angle to maintain the neck‐shaft angle. The nonthreaded dynamic rod is located at the mid‐axis of the femoral neck, forming an angle of 7.5° with the anti‐rotation screw to achieve angular stability. However, the anti‐rotation screw is the only FNS component that holds cancellous bone, and the FNS placement is in the femoral neck axis, which cannot effectively resist rotational forces. At the same time, the cancellous bone surrounding the FNS component is prone to collapse, while the three screws in ICCS + TCS are closer to the cortical bone, thus exhibiting smaller deformation in the cyclic pressure test. However, FNS exhibits higher axial stiffness and displacement in static compression, which is consistent with the results of FEA. This may be attributed to its thicker power rods and stress dispersion capability.

### 4.2 Clinical Research Verification

In clinical studies, the operation of ICCS + TCS is considered minimally invasive, featuring smaller surgical incisions, less intraoperative blood loss, and less trauma to patients. Yin et al. [62] research suggests that if the femoral neck shortening is greater than 5 mm, varying degrees of hip joint pain and limited abduction function may occur. If the shortening exceeds 10 mm, symptoms such as pain and limping may appear, affecting hip joint function and quality of life. In the experiment, the reduced length of the ICCS + TCS group was significantly smaller than the aforementioned standard. At the final follow‐up, significant improvements were observed in Harris score, internal–external rotation ROM of the hip, and flexion‐extension ROM of the hip. This may be attributed to the adequate biomechanical performance provided by ICCS + TCS, which ensures initial stability of the fracture ends postoperatively, creates a favorable environment for fracture healing, promotes vascular regeneration in the femoral neck and fracture healing, enables patients to shorten their bed rest time, undergo early lower limb rehabilitation training, effectively restore hip joint function, and reduce related complications.

ICCS + TCS has good stability, but it fails to fully address the potential increased surgical risk. First, ICCS + TCS is more complex than traditional CCS or FNS solutions and requires a longer learning curve. In the process of ICCS + TCS fixation, hollow compression screw guide pin placement needs to form a spatial configuration of three parallel inverted triangular guide pins and one transverse vertical fracture line guide pin, and the problem of mutual obstruction between screws should be considered when hollow screw placement, which is relatively difficult, time‐consuming, and has many fluoroscopic times.

However, the ICCS + TCS approach still carries potential surgical risks. Firstly, ICCS + TCS is more complex than traditional CCS or FNS protocols, necessitating a longer learning curve. During the fixation process of ICCS + TCS, the placement of cannulated screws requires the formation of a spatial configuration consisting of three parallel inverted triangular guide wires and one transverse vertical fracture line guide wire. Additionally, when inserting the cannulated screws, the issue of mutual obstruction between screws must be considered, making the surgery relatively difficult, time‐consuming, and requiring multiple X‐ray fluoroscopy procedures. However, the ICCS + TCS approach can provide stronger stability to the fracture ends, create a stable mechanical environment for fracture healing, and enhance the likelihood of fracture healing. For special types of patients, such as those with osteoporosis, the ICCS + TCS approach may pose challenges due to the hollow compression screw's mechanism, which involves compressing cancellous bone. This necessitates a high quality of bone at the fracture site. Patients with osteoporosis often experience significant bone loss, making it difficult for the hollow compression screw to provide effective fixation. Consequently, there is a higher likelihood of internal fixation failure and screw backout. Therefore, for young adults and elderly patients without osteoporosis, the ICCS + TCS fixation approach may be more suitable.

### 4.3 Limitations and Prospects

In recent years, with the rapid development of electronic computers and medical imaging, FEA, as a relatively emerging theoretical research method, has been widely applied not only in engineering applications and materials science, but also in medical‐related research fields, especially in orthopedics. This study simulates P3FNFs using FEA and BT, and compares the biomechanical properties of ICCS + TCS and FNS to complete theoretical verification. At the same time, clinical research is combined to support the effectiveness of the new scheme.

This experiment also has some shortcomings: (1) FEA fails to fully simulate the structure of the actual femoral neck. The model is based on the assumption that the femur is a homogeneous, continuous, and isotropic elastic material, but in reality, human bones are anisotropic materials. When conducting experiments with FNS as a single entity, the characteristics of its multiple components are not fully reflected. Therefore, the material properties used in finite element experiments may affect the final results. However, the goal of FEA is to study overall trends rather than precise values, so such a setup can be considered reasonable. (2) Artificial composite bone (Synbone LD2350, Left, Switzerland) cannot fully reflect the actual bone tissue conditions, and there are differences compared to actual clinical use. It cannot represent some special bone types (such as osteoporosis patients). Therefore, in future research, we will improve the experiment using human specimens. (3) The surgical difficulty of ICCS + TCS is relatively high, requiring advanced surgical skills and multiple intraoperative fluoroscopy procedures. Additionally, the sample size in this experiment is small and the follow‐up period is relatively short; it may not be sufficient to evaluate long‐term complications such as osteonecrosis of the femoral head. Therefore, large‐scale multicenter clinical studies are needed for verification. For posterior and posteromedial bone defects caused by comminuted femoral neck fractures, the use of CCS, which relies on compressive fixation of bone (requiring a high bone mass), is relatively limited. Therefore, this article focuses on young adults (who are relatively less prone to bone defects). However, in response to the reviewer's comment on complex types of femoral neck fractures, our team will conduct further research and targeted discussions in the later stages. (4) The clinical study was not randomized (potential selection bias may exist). (5) The finite element analysis did not simulate muscle attachment (such as the tensile forces of the gluteus medius and iliopsoas muscles), resulting in deviations from the actual human hip joint. At the same time, we will continue to delve deeper into the research on the ICCS + TCS fixation protocol, encompassing the increase in screw diameter, screw implantation position and angle, as well as comparisons with other current clinical internal fixation systems for treating femoral neck fractures.

## 5 Conclusion

In summary, this experiment explored the biomechanical performance of ICCS + TCS in the fixation of Pauwels type III femoral neck fractures in young adults through finite element analysis and in vitro biomechanical experiments. It also compared this performance with that of FNS and retrospectively analyzed the clinical treatment effect of ICCS + TCS. The results indicate that ICCS + TCS exhibits biomechanical properties similar to FNS in the treatment of P3FNFs, while possessing stronger torsional stiffness and lower overall deformation. Clinically, it has also achieved relatively good therapeutic effects, providing a theoretical basis for subsequent research and offering a reference for the treatment of P3FNFs.

## Author Contributions

**Yujie Li:** conceptualization, software, methodology, validation, writing – original draft. **Zhenyu Wen:** writing – original draft. **Naiqiang Zhuo:** writing – review and editing, visualization, project administration. **Zhang Jian:** writing – original draft, supervision, resources, data curation. **Shi Shen:** writing – review and editing, visualization, formal analysis.

## Funding

This study was sponsored by the Special scientific research project of Sichuan Provincial Medical Science and Technology Innovation Research Association (2025YCZD95), the Natural Science Foundation of Sichuan Province (2024NSFSC0677), the Southwest Medical University university‐level project (2019ZQN094), and the Luzhou‐Southwest Medical University Joint Proiect (2020LZXNYDJ06).

## Ethics Statement

This study was approved by the Ethics Committee of the Affiliated Hospital of Southwest Medical University (Ethics Approval Number: KY2023318). All patients provided informed consent for the treatment plan and voluntarily participated in this study.

## Consent

The authors have nothing to report.

## Conflicts of Interest

The authors declare no conflicts of interest.

## Data Availability

Data sharing not applicable to this article as no datasets were generated or analysed during the current study.
